# Community composition and spatial aggregation patterns of the endangered endemic plant *Menonvillea linearifolia* (Brassicaceae) in the Atacama Desert

**DOI:** 10.3897/BDJ.14.e185999

**Published:** 2026-03-26

**Authors:** Carlos E. Valdivia, Mauricio A. Cisternas

**Affiliations:** 1 Laboratorio de Vida Silvestre, Departamento de Ciencias Biológicas y Biodiversidad, Universidad de Los Lagos, Osorno, Chile Laboratorio de Vida Silvestre, Departamento de Ciencias Biológicas y Biodiversidad, Universidad de Los Lagos Osorno Chile; 2 Instituto de Investigaciones Agropecuarias, INIA, La Cruz, Chile Instituto de Investigaciones Agropecuarias, INIA La Cruz Chile

**Keywords:** *Menonvillea minima*, Chile, flowering desert, spatial aggregation, sandy habitats

## Abstract

*Menonvillea linearifolia* is an endangered annual herb endemic to north-central Chile, growing on sandy coastal slopes within the Atacama Desert flowering zone. To characterise its ecological context, we surveyed the floristic composition, plant cover and spatial aggregation patterns of its associated plant community in Punta de Choros, Coquimbo Region, during a flowering desert event. Using 20 transects and 1,000 microplots, we recorded 29 vascular plant species, with a dominance of annual herbs (44.8%) and high endemism (79.3%). The community exhibited strong dominance of *Cistanthe longiscapa*, whereas most species occurred at low cover and in scattered patches. Spatial analyses (variance-to-mean ratio, Morisita, Green and negative binomial indices) revealed predominantly aggregated distributions. In particular, *Menonvillea linearifolia*, *Alstroemeria werdermannii*, *Echinopsis deserticola* and *Heliotropium floridum* were highly aggregated, reflecting limited dispersal and dependence on specific microhabitats. These traits make *M. linearifolia* particularly vulnerable to disturbances, including off-road vehicle use, grazing, mining expansion, and climatic variability. The results emphasize the importance of protecting key microhabitats and incorporating spatial structure into conservation strategies. This study provides the first quantitative ecological baseline for *M. linearifolia*, supporting targeted habitat management and conservation actions in the Atacama flowering desert ecosystems.

## Introduction

The Atacama Desert, located in northern Chile, is amongst the oldest and most extreme arid environments on Earth. It is characterised by hyperaridity and highly irregular precipitation regimes with extremely low annual rainfall (Rundel et al. 1991, Squeo et al. 2018). Despite these harsh conditions, the desert supports a uniquely diverse and highly specialised flora, marked by elevated levels of endemism and specialised ecophysiological strategies to withstand severe water stress and nutrient-poor soils (Arroyo et al. 1998, del Río et al. 2021, Luebert and Pliscoff 2023). Key anthropogenic threats include habitat loss and fragmentation driven by mining and energy development, chronic disturbance from roads and off-road vehicles and competing demands for scarce water resources, which, together, can reduce population connectivity and degrade microsites critical for recruitment in desert plants ([Bibr B13947820][Bibr B13946497][Bibr B13946514]). Therefore, this region represents a natural laboratory for studying biological adaptation and ecological resilience under extreme climatic conditions, but it is also highly sensitive to environmental fluctuations and anthropogenic pressures (Rundel et al. 1991, Squeo et al. 2018, Humaña and Valdivia 2024).

A remarkable ecological phenomenon in this region is the so-called "flowering desert", which occurs in years associated with unusually high rainfall, often linked to El Niño–Southern Oscillation (ENSO) events (Squeo et al. 2006, Squeo et al. 2018). These rains trigger the massive germination of annual species and the synchronised flowering of numerous perennial plants, transforming extensive desert areas into floristic communities with high cover and species richness (Squeo et al. 2006, Squeo et al. 2018). Recent remote-sensing studies have provided quantitative evidence of this dynamic, revealing that flowering desert events can cover thousands of square kilometres and last for several months (Chávez et al. 2019). Major events have been recorded in 1997–1998, 2002–2003 and 2011 (Chávez et al. 2019), with additional events documented in 2015, 2017, 2021, 2022 and 2025 (Araya et al. 2020, Astorga-Eló et al. 2020, Martínez-Harms et al. 2022, Rozas-Lazcano et al. 2025). Notably, the shorter return intervals observed since the mid-2010s suggest that "flowering desert" events may be becoming increasingly recurrent, likely reflecting climate-change–driven shifts in precipitation extremes and the frequency and/or intensity of ENSO-related rainfall anomalies (Chávez et al. 2019). This ephemeral, yet intense, dynamic sustains key ecological interactions, including pollination networks, nutrient flows and resource provisioning for insects and vertebrates, representing a critical event for the persistence of numerous native species (Squeo et al. 2006, Squeo et al. 2018, Humaña et al. 2019, Humaña et al. 2021).

However, this exceptional biodiversity faces increasing threats from mining expansion, climate change, overgrazing, the introduction of exotic species and habitat fragmentation, which have led several endemic species of the flowering desert to be classified under concerning conservation categories (Squeo et al. 2001, Ministerio de Medio Ambiente 2023). One emblematic example is *Bomarea ovallei* (Phil.) Ravenna (= *Leontochir ovallei* Phil., Alstroemeriaceae), a conspicuous and charismatic geophyte with striking red flowers that has become an icon of the Atacama Desert bloom. This species is endemic to a narrow range in the Atacama Region and its populations have declined due to habitat loss, illegal collection and the expansion of agricultural and mining activities, leading to its classification as endangered in Chile (Squeo et al. 2001, Hoffmann et al. 2015, Ministerio de Medio Ambiente 2023).

In contrast, other threatened taxa of the flowering desert are far less conspicuous and poorly studied. A clear example is *Menonvillea linearifolia* (Hook. & Arn.) Al-Shehbaz & Salariato (= *M. minima* Rollins, Brassicaceae), an inconspicuous annual herb endemic to the southern Atacama and northern Coquimbo Regions of Chile. This species grows on sandy slopes and plains where flowering is concentrated during spring following winter rainfall events (Ministerio de Medio Ambiente 2023). *Menonvillea linearifolia* has small, discontinuous populations, is highly sensitive to interannual precipitation variability and faces multiple pressures from habitat degradation and land-use change. These factors have led to its classification as Endangered (Ministerio de Medio Ambiente 2023), a category originally assigned under its previous name *M. minima* (Ministerio de Medio Ambiente 2023, Al-Shehbaz and Salariato 2024).

These cases exemplify how both emblematic and inconspicuous species of the flowering desert are increasingly exposed to pressures that threaten their persistence, underscoring the urgency of generating robust ecological knowledge to guide conservation strategies and management actions in this unique region. In this context, studying the associated flora of threatened species is essential for understanding their ecological niche, identifying habitat requirements, assessing the structure of the communities in which they occur and generating inputs for *in situ* conservation strategies (Franklin et al. 2002, Sutherland et al. 2004). Such information is particularly valuable for species for which little or no ecological data are available. In the specific case of *M. linearifolia*, no studies have yet documented its associated vegetation or community structure. Characterising the vegetation associated with this species can, therefore, provide key insights into patterns of floristic co-occurrence, favourable environmental conditions and potential habitat indicator species (Franklin et al. 2002, Squeo et al. 2018). Accordingly, this study aims to qualitatively and quantitatively describe the accompanying vegetation of *M. linearifolia* in the northern Coquimbo Region through vegetation cover transects, to provide ecological background information that contributes to its conservation (Ministerio de Medio Ambiente 2023).

## Material and methods

**Study site and species**

**Figure 1.**
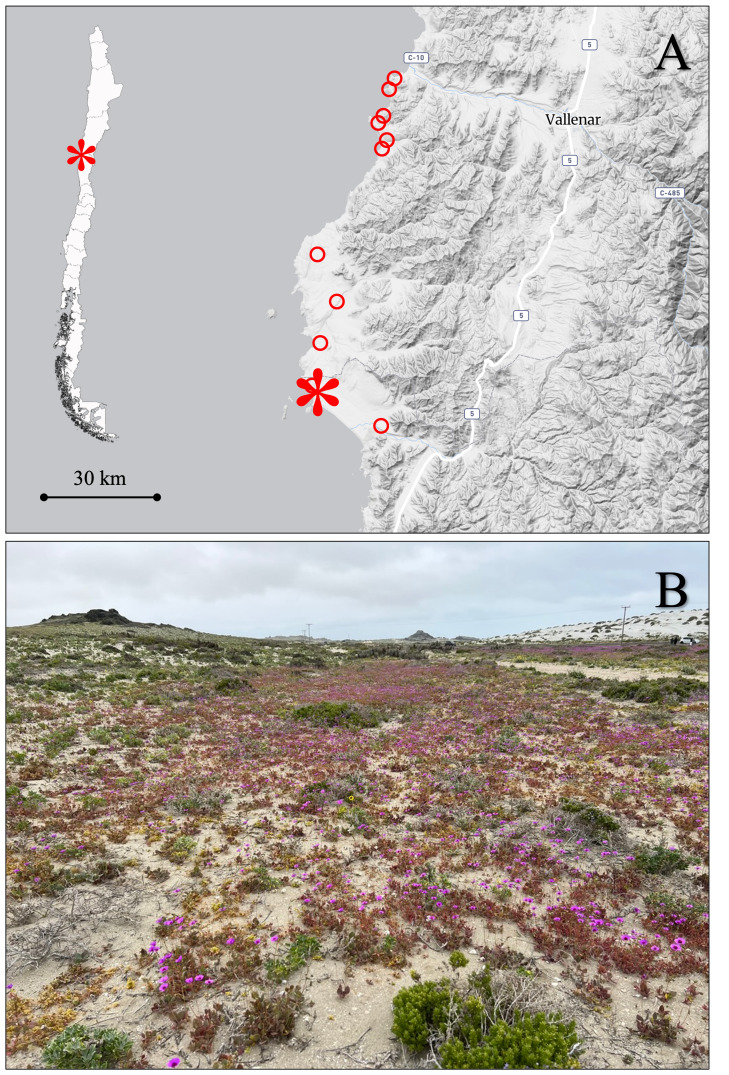
Study area and typical vegetation at the *Menonvillea linearifolia* site during a “flowering desert” year. A) Relief map of the study area, with red circles indicating occurrence records of *M. linearifolia* sourced from iNaturalist (www.inaturalist.org) and a red asterisk marking the study site. The base map was generated from a screenshot of Macrostrat (https://macrostrat.org) and edited in PowerPoint; B) Vegetation at the study site during a “flowering desert” year, showing extensive ephemeral herbaceous cover on sandy coastal substrates. Photo credit: Carlos E. Valdivia. No copyrighted or third-party graphics were used.

The study was conducted in October 2025 at a locality situated at approximately 29°13’ S, 71°27’ W, in the northern portion of the Coquimbo Region, Chile, near the coastal village of Punta de Choros (Fig. 1). This site is located along the coastal margin of the Atacama Desert and corresponds to the vegetation belt classified by Luebert and Pliscoff (2023) as the *Coastal Mediterranean Desert Shrubland of Oxalis virgosa* Molina and *Heliotropium stenophyllum* Hook. & Arn. The area has a Mediterranean-type climate strongly influenced by the Pacific Ocean, with numerous cloudy days and frequent morning fog and generally mild air temperatures with a narrow thermal range due to oceanic proximity (Valdivia et al. 2011). Interannual rainfall is highly irregular, with dry years averaging < 25 mm of annual precipitation and wet years exceeding 175 mm, occurring in irregular cycles that have been linked to the ENSO episodes (Valdivia et al. 2011). This vegetation unit is characterised by open, low stature shrublands dominated by the xerophytic shrubs *H. stenophyllum* and *O. virgosa*, with frequent participation of *Flourensia thurifera* (Molina) J.C. Ospina & S.E. Freire, *Nolana coelestis* (Lindl.) Miers ex Dunal, *N. crassulifolia* Poepp. and *Encelia canescens* Lam. On coastal terraces, *Haplopappus cerberoanus* (J. Rémy) Reiche is common, whereas on hill slopes, it is replaced by *H. pulchellus* DC. and *H. parvifolius* (DC.) Gay (Luebert and Pliscoff 2023). During the spring of rainy years, the herbaceous layer is enriched by ephemeral annuals that form a dense and colourful cover on sandy soils, including *Cryptantha glomerata* Lehm. ex Fisch. & C.A. Mey., *Cistanthe coquimbensis* (Barnéoud) Carolin ex Hershk., *Chorizanthe glabrescens* Benth., *Erodium cicutarium* (L.) L'Hér. ex Aiton and other native and introduced taxa. This phenomenon reflects both the ecological response to episodic precipitation and, in some cases, disturbance signals linked to anthropogenic activities (Luebert and Pliscoff 2023). The terrain at the site consists of gently sloping sandy plains formed by aeolian deposits, with coarse, nutrient-poor soils. The prevailing bioclimate is thermomediterranean to inframediterranean, with marked water deficits throughout most of the year and irregular rainfall events associated with ENSO episodes, which trigger massive germination and flowering events (Luebert and Pliscoff 2023).

**Figure 2.**
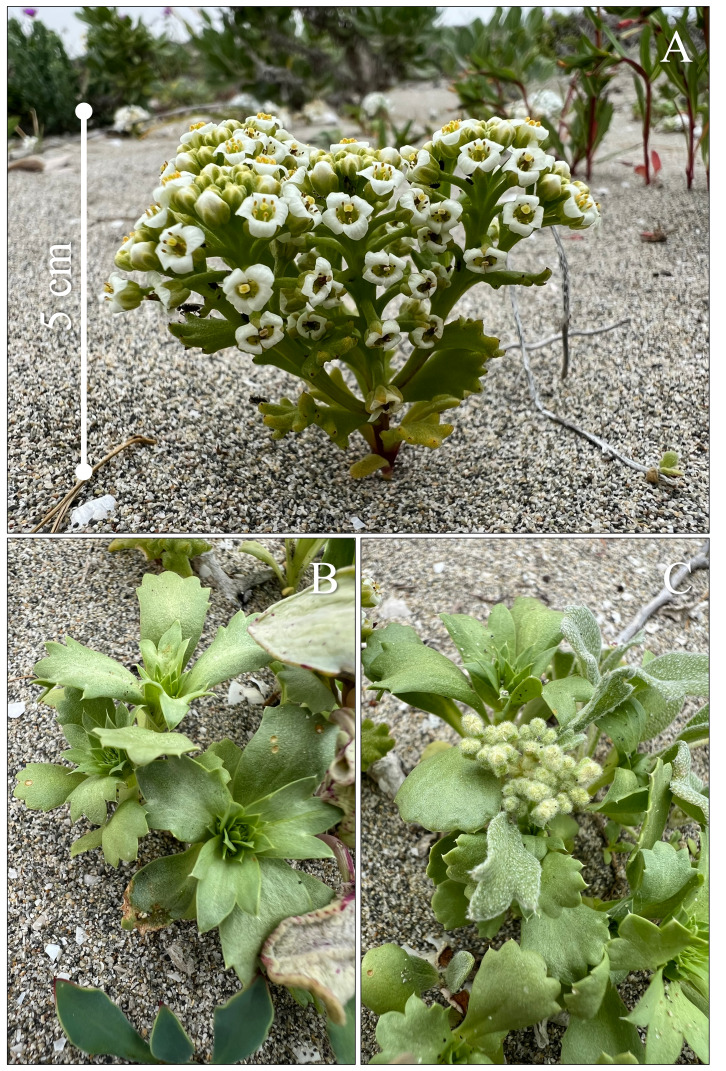
*Menonvillea linearifolia* (Brassicaceae), an endangered annual herb, in different phenological stages at Punta de Choros, Coquimbo Region, Chile: **A** flowering stage; **B** vegetative stage; **C** budding stage. Photo credits: Carlos E. Valdivia.

*Menonvillea linearifolia* (Brassicaceae) is a small annual herb, endemic to north-central Chile, restricted to sandy slopes and plains in areas influenced by episodic spring blooms. It produces small, white flowers and forms low, compact rosettes that allow it to withstand extreme aridity and wind exposure (Fig. 2). Its phenology is closely tied to rainfall events, with rapid germination and flowering during years with sufficient precipitation. The species typically occurs in sparse populations and is often interspersed amongst other annual herbs, making it less conspicuous than many co-flowering taxa. Due to its narrow distribution, fragmented populations and sensitivity to climatic variability, *M. linearifolia* is currently classified as Endangered in Chile (Ministerio de Medio Ambiente 2023).

**Floristic composition and cover**

Plant species and substrate cover were recorded using a modified point-intercept method with circular microplots along transects established within the study site. A total of 20 transects, each 50 m in length and spaced at least 5 m apart, were laid out over representative areas of the *M. linearifolia* population. At 1 m intervals along each transect, we recorded a single point-intercept within a 5 cm-radius circular microplot (78.5 cm²) centred on the sampling point, scoring the dominant plant species (or substrate type). Species ‘cover’ was then expressed as the percentage of microplots (hits) assigned to each species across transects, allowing for a more robust representation of the local floristic composition and microspatial heterogeneity than would be achieved through a single intercept point. This sampling design, commonly used in arid ecosystems, yielded a total of 1,000 sampling plots across the site (Mueller-Dombois and Ellenberg 1974, Elzinga et al. 1998, Godínez-Alvarez et al. 2009, Bonham 2013).

To evaluate whether the sampling effort was sufficient to capture local floristic diversity, a rarefaction analysis was performed (Gotelli and Colwell 2001, Colwell et al. 2004). Species accumulation curves were generated in R using the package *vegan* (function specaccum), based on the transect × species presence–absence matrix and an iterative random resampling of transects without replacement (method = “random”, 1,000 permutations) to estimate expected species richness and 95% confidence intervals (Gotelli and Colwell 2001, Magurran 2004). The R script and input matrix format are provided as Supplementary Material (Suppl. materials 2, 1). The resulting curve was used to assess whether the observed sampling effort approached an asymptote, indicating sufficient coverage of local plant diversity (Magurran 2004, Colwell et al. 2012, Hsieh et al. 2016).

**Spatial aggregation patterns**

To evaluate the spatial distribution patterns of each plant species, we calculated several spatial aggregation indices, based on the number of occurrences per transect. All spatial aggregation indices and classification were computed in R using a fully reproducible script that automates index calculation and category assignment from the transect-level occurrence matrix. The complete R code and input data format are provided as Supplementary Material (Suppl. material 2). The indices and their corresponding formulas are as follows:

1. Variance-to-mean ratio (I)

I = s² / x̄

where s² is the sample variance and x̄ is the sample mean. This index measures departures from randomness: I ≈ 1 indicates random distribution, I > 1 aggregation and I < 1 uniformity (Elliott 1979, Krebs 1999).

2. Morisita’s index (Id)

Id = [n Σ x_i_(x_i_ - 1)] / [X(X - 1)]

where x_i_ is the number of individuals in sample i, n is the number of samples and X is the total number of individuals. This index quantifies aggregation intensity independent of sample size: Id ≈ 1 random, Id > 1 aggregated, Id < 1 uniform (Morisita 1962, Krebs 1999).

3. Green’s index (G)

G = (I - 1) / (Σx - 1)

This index standardises aggregation between 0 and 1, allowing comparisons across species. G < 0.1 indicates weak aggregation, 0.1 ≤ G ≤ 0.3 moderate aggregation and G > 0.3 strong aggregation (Green 1966, Ludwig and Reynolds 1988).

4. Negative binomial parameter (k)

k = x̄² / (s² - x̄)

This parameter estimates clumping intensity: k > 8 suggests random or uniform distribution, 1 ≤ k ≤ 8 moderate aggregation and k < 1 strong aggregation (Elliott 1979, Perry 1995).

Species were classified into four categories, based on these indices: highly aggregated (Id > 1, G > 0.3, k < 1), moderately aggregated (Id > 1, 0.1 ≤ G ≤ 0.3, 1 ≤ k ≤ 8), weakly aggregated (Id > 1, G < 0.1, k > 8) and random/uniform (Id ≈ 1 or Id < 1, G ≈ 0, k very large). Species with low numbers of occurrences (≤ 5) were flagged with an asterisk (*) in the results table, indicating that their spatial aggregation patterns should be interpreted with caution because low sample size may inflate index values.

All vascular plant species were identified in the field or subsequently confirmed using herbarium and photographic references. Taxonomic nomenclature, growth form and origin of each plant species followed the Catálogo de las Plantas Vasculares de Chile (Rodriguez et al. 2018).

## Results

**Floristic composition and cover**

**Figure 3.**
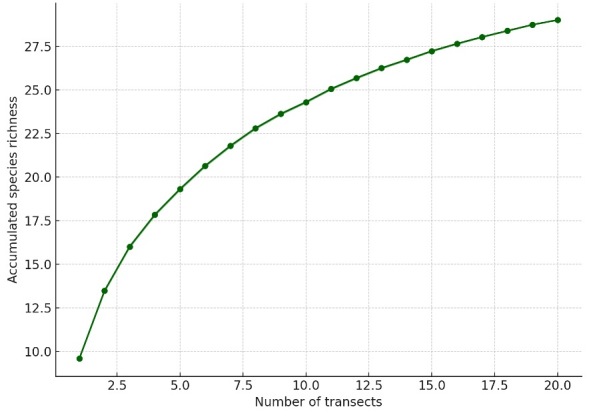
Sample-based rarefaction curve showing the expected accumulation of plant species richness with increasing sampling effort (number of transects) in Punta de Choros. The solid line represents the mean richness estimated from 1,000 random permutations of transect order. The standard error was very small (0.1 species at maximum sampling effort) and, thus, not visually distinguishable at the scale of the graph, reflecting the high consistency of the species accumulation pattern across transects.

A total of 29 vascular plant species were recorded within the study area, most of them annual herbs typical of the flowering desert ecosystem (Table 1). The community was dominated by annual herbs (13 species; 44.8%), followed by shrubs (7 species; 24.1%), perennial herbs (7 species; 24.1%), whereas subshrubs (1 species; 3.4%) and one succulent shrub (3.4%) were comparatively rare. Regarding their origin, most species were endemic (23 species; 79.3%), with five native species (17.2%) and only one introduced species (3.4%). Species accumulation analysis indicated that the sampling effort was sufficient to capture most of the local floristic diversity, as the rarefaction curve reached an asymptote after approximately 15 transects (Fig. 3).

**Figure 4.**
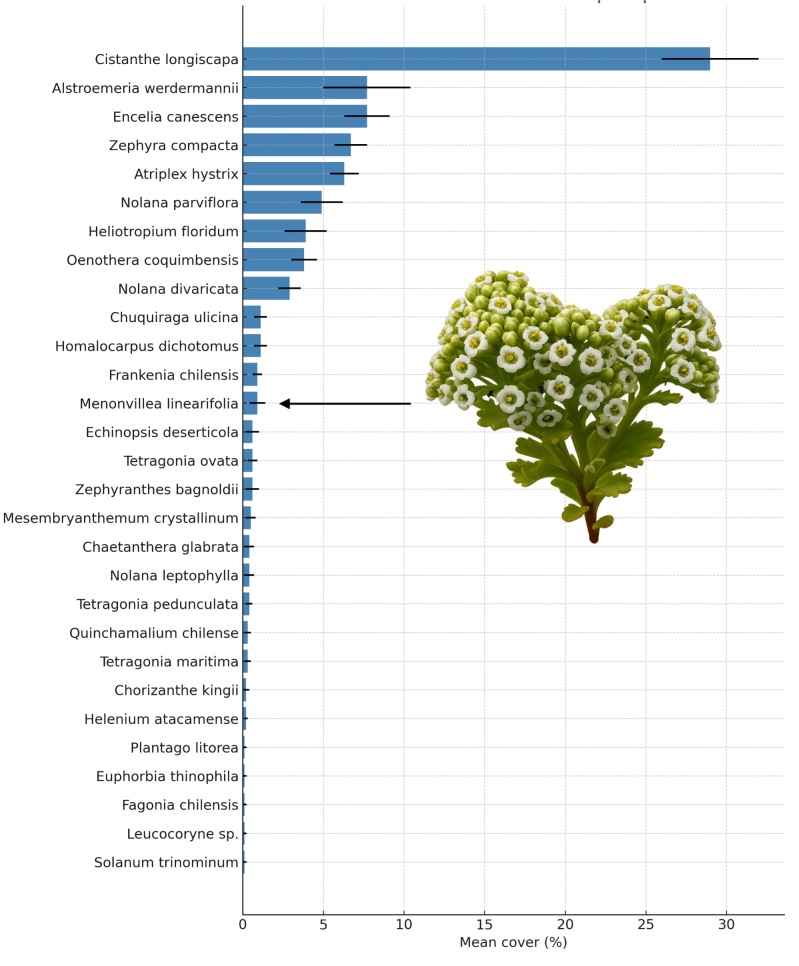
Mean percentage cover (± standard error) of plant species recorded in the 20 transects surveyed at Punta de Choros. Species are arranged from highest to lowest mean cover. *Menonvillea linearifolia* was amongst the least dominant species, reflecting its infrequent occurrence within the study site.

A small set of species dominated community cover (five species with mean cover > 5%), whereas most taxa contributed little to total cover (24 species with mean cover < 5%) (Fig. 4). *Cistanthe longiscapa* accounted for the highest average cover, representing a conspicuous component of the herbaceous layer throughout the study site. Other abundant species included *Alstroemeria werdermannii*, *Atriplex hystrix* and *Zephyra compacta*, all of which displayed widespread distributions, but variable cover across transects.

High mean cover values were concentrated in a small set of dominant species (five species with mean cover > 5%), including *Cistanthe longiscapa*, *Alstroemeria werdermannii*, *Encelia canescens*, *Zephyra compacta* and *Atriplex hystrix* (Fig. 4). Intermediate mean cover values (≈ 3–5%) were observed for taxa such as *Nolana parviflora*, *Heliotropium floridum* and *Oenothera coquimbensis* (Fig. 4). Transect-level occurrence data from the presence–absence matrix (Suppl. material 1) further indicate that several of these non-dominant taxa were present in only a subset of transects, consistent with spatial heterogeneity across the site. In contrast, most species, including several endemic taxa, such as *Chaetanthera glabrata*, *Chorizanthe kingii* and *Homalocarpus dichotomus*, showed low cover and restricted occurrences, characteristic of low-density populations, adapted to microhabitats with specific conditions.

Bare substrate represented a substantial fraction of the surveyed area, reflecting the open structure typical of this coastal desert ecosystem and the strong spatial heterogeneity in plant establishment. This dominance of open ground interspersed with patches of vegetation is consistent with the episodic and irregular nature of flowering desert events.

**Spatial aggregation patterns**

Spatial aggregation analyses revealed a predominance of non-random spatial distributions amongst plant species. The indices calculated (variance-to-mean ratio I, Morisita’s index Id, Green’s index G and the negative binomial parameter k) showed that most species exhibited aggregated patterns of varying intensity (Table 2).

High aggregation was observed for *Alstroemeria werdermannii*, *Echinopsis deserticola*, *Heliotropium floridum* and *Menonvillea linearifolia*. Several low-frequency taxa, such as *Chorizanthe kingii*, *Chaetanthera glabrata* and *Mesembryanthemum crystallinum*, also showed high aggregation values, although these results must be interpreted with caution due to their low number of occurrences (≤ 5). Moderate aggregation characterised common species, such as *Atriplex hystrix*, *Chuquiraga ulicina*, *Homalocarpus dichotomus*, *Nolana divaricata*, *Oenothera coquimbensis*, *Tetragonia ovata* and *Zephyra compacta*. In contrast, *Cistanthe longiscapa*, *Encelia canescens* and *Nolana parviflora* exhibited weak aggregation, suggesting more dispersed or near-random distributions. Only *Helenium atacamense* presented an approximately random or uniform spatial pattern.

Overall, the aggregated distribution observed for most species reflects the patchy nature of plant establishment in this coastal desert ecosystem, shaped by irregular precipitation, microtopography and edaphic heterogeneity. In particular, the marked aggregation pattern of *M. linearifolia* suggests a dependence on specific microhabitat conditions and localised dispersal.

## Discussion

The floristic composition of the plant community associated with *Menonvillea linearifolia* reflects the characteristic structure of ephemeral vegetation in the Atacama Desert during flowering years, with a dominance of annual herbs and a high proportion of endemic species (Squeo et al. 2006, Squeo et al. 2018, Martínez-Harms et al. 2022). This composition is also consistent with pulse-driven dynamics described for the southern Atacama, where rainfall events trigger rapid vegetation change and sequential flowering through time, reshaping community structure over short temporal windows (Vidiella et al. 1999). The observed richness is consistent with previous studies conducted in coastal sectors of the Atacama and Coquimbo Regions during flowering desert events, where species assemblages typically include a small number of abundant taxa and many low-frequency species restricted to specific microhabitats (Rundel et al. 1991, Squeo et al. 2018). The high proportion of annual herbs is indicative of a community that responds rapidly to episodic rainfall pulses, germinating and completing its life cycle within a single season (Squeo et al. 2006), a pattern matching the rapid post-rain shifts and phenological sequencing reported for desert flowering assemblages (Vidiella et al. 1999). By contrast, shrubs and perennial herbs account for a smaller proportion of the flora, reflecting the persistence of structural elements even in years with low precipitation (Rundel et al. 1991, Schwinning and Sala 2004).

The dominance of *C. longiscapa* in terms of mean cover is consistent with its known ecological strategy as a widespread and fast-growing annual capable of taking advantage of wet years to form dense patches across sandy plains (Squeo et al. 2006, Squeo et al. 2018). Other abundant species, such as *A. werdermannii*, *A. hystrix* and *Z. compacta*, also contribute substantially to the herbaceous layer, while many species remain patchy and infrequent, likely reflecting their dependence on microsite availability and local environmental filters (Rundel et al. 1991, Luebert and Pliscoff 2023). The large proportion of bare substrate observed further underscores the open structure of these desert communities and their strong sensitivity to climatic variability (Squeo et al. 2006, Squeo et al. 2018). This pattern also aligns with evidence that Atacama plant assemblages can exhibit strong spatial heterogeneity and shifting associations across spatial scales under arid conditions (Carvajal et al. 2023).

The spatial aggregation analyses revealed that most species exhibited non-random distributions, with a predominance of aggregated patterns of varying intensity (Morisita 1962, Green 1966, Perry 1995). These results are consistent with other studies in arid and semi-arid ecosystems, where plant distributions are typically patchy due to the combined influence of water availability, microtopography, soil texture and biotic interactions, such as facilitation and dispersal limitation (Schwinning and Sala 2004, Godínez-Alvarez et al. 2009). In the Atacama Desert specifically, functional diversity and spatial association analyses across spatial scales have shown that community assembly patterns can remain broadly stable along aridity gradients, while species associations still exhibit scale-dependent structure (Carvajal et al. 2023).

Highly aggregated species, such as *A. werdermannii*, *E. deserticola*, *H. floridum* and *M. linearifolia*, exhibit fruit and dispersal traits that likely contribute to their clumped spatial patterns. *Alstroemeria werdermannii* produces dehiscent capsules, *H. floridum* produces nutlets and *M. linearifolia* has winged, dehiscent siliques, all of which are dispersed mainly by gravity (barochory), resulting in seeds remaining close to the parent plant. *Echinopsis deserticola*, in contrast, bears fleshy and aromatic dehiscent berries that are animal-dispersed (endozoochory), which can also lead to clustered seed deposition in localised microsites (Green 1966, Perry 1995). Such trait-mediated dispersal constraints can reinforce patch formation and local co-occurrence patterns detected at multiple spatial scales in Atacama Desert communities (Carvajal et al. 2023).

The aggregation pattern of *M. linearifolia* is particularly relevant for conservation, as it indicates that its populations occur in discrete patches rather than being evenly distributed across the landscape (Perry 1995). This pattern is typical of many rare and endemic desert species and suggests that their persistence may depend on the maintenance of specific microhabitats, such as sheltered sandy patches with adequate soil moisture (Rundel et al. 1991, Squeo et al. 2001, Squeo et al. 2006). This localised distribution may increase the species’ vulnerability to multiple threats, including habitat degradation caused by off-road vehicle traffic and livestock grazing, fragmentation resulting from the expansion of energy and mining projects in the region and climate variability that affects the frequency and intensity of flowering events (Rundel et al. 1991, Squeo et al. 2001, Squeo et al. 2006, Squeo et al. 2018). Moreover, because vegetation change and flowering can occur sequentially after rainfall pulses, recruitment opportunities may be concentrated in short, temporally structured windows following rain events (Vidiella et al. 1999), potentially increasing demographic sensitivity to shifts in precipitation timing and magnitude. From a conservation perspective, these results highlight the need to protect and manage key microhabitats where the species is concentrated, as well as to incorporate spatial aggregation patterns into habitat restoration and population reinforcement programmes. As the species has dehiscent, gravity-dispersed fruits, its natural colonisation capacity is limited, which reinforces the importance of *in situ* conservation and targeted translocation to suitable habitats.

From a broader perspective, these results highlight the importance of combining floristic, structural and spatial pattern analyses when characterising plant communities associated with threatened species (Green 1966, Perry 1995). Identifying dominant and co-occurring taxa, as well as their spatial patterns, provides essential ecological information that can guide habitat management and restoration efforts (Squeo et al. 2018). Recent work in the Atacama further supports the value of integrating functional diversity with spatial association analyses across scales to interpret community structure under aridity (Carvajal et al. 2023). In the case of *M. linearifolia*, conservation actions should prioritise maintaining and protecting its patchy microhabitats, minimising disturbances and monitoring interannual dynamics linked to rainfall variability (Squeo et al. 2006, Rozas-Lazcano et al. 2025). Such monitoring should also consider post-rain vegetation turnover and sequential flowering dynamics that can modulate habitat suitability within and amongst seasons (Vidiella et al. 1999).

## Supplementary Material

CF29943E-C5E4-59FC-89B9-18B5402EBD94 10.3897/BDJ.14.e185999.suppl1 Supplementary material 1 Community Composition and Spatial Aggregation Patterns of the Endangered Endemic Plant Menonvillea linearifolia (Brassicaceae) in the Atacama Desert Data type Matrix and Workflow for species accumulation curves Brief description Appendix S1 provides a fully reproducible workflow to generate species accumulation (rarefaction) curves from the study’s presence–absence matrix. It reads the Excel file (“Matriz presencia ausencia.xlsx”), converts the data to a transect × species matrix and computes accumulation curves in R using vegan::specaccum with random resampling of transects without replacement (1,000 permutations) and 95% confidence intervals. The appendix also exports the curve values (CSV) and a publication-ready figure (PNG) and includes the input matrix used in the analyses. File: oo_1553577.pdf https://binary.pensoft.net/file/1553577 Carlos E. Valdivia, Mauricio A. Cisternas

2A6171EF-2EA8-5F6E-8B7F-B9CCF1BC1471 10.3897/BDJ.14.e185999.suppl2 Supplementary material 2 Community Composition and Spatial Aggregation Patterns of the Endangered Endemic Plant Menonvillea linearifolia (Brassicaceae) in the Atacama Desert Data type Script/workflow for index calculation Brief description Appendix S2 provides a fully reproducible R workflow to compute the spatial aggregation indices used in the manuscript — variance-to-mean ratio (I), Morisita’s index (Id), Green’s index (G) and the negative binomial aggregation parameter (k) — from the transect-level occurrence matrix (rows = species, columns = transects). It then assigns each species to the four aggregation categories defined in the Methods and flags species with ≤ 5 total occurrences with an asterisk, exporting the complete results table as a CSV file File: oo_1553578.pdf https://binary.pensoft.net/file/1553578 Carlos E. Valdivia, Mauricio A. Cisternas

## Figures and Tables

**Table 1.** List of vascular plant species recorded within the *Menonvillea linearifolia* population study area. For each species, the taxonomic family, growth form, origin (endemic, native or introduced), fruit type, and main dispersal agent are indicated. Taxonomic nomenclature follows Rodriguez et al. (2018).

Species	Family	Growth form	Origin	Fruit type	Main dispersal agent
*Alstroemeria werdermannii* Ehr. Bayer	Alstroemeriaceae	Perennial herb	Endemic	Dehiscent capsule	Gravity
*Atriplex hystrix* Phil.	Amaranthaceae	Shrub	Endemic	Utricle	Wind
*Chaetanthera glabrata* (DC.) F. Meigen	Asteraceae	Annual herb	Endemic	Achene	Wind
*Chorizanthe kingii* Phil.	Polygonaceae	Subshrub	Endemic	Achene	Wind
*Chuquiraga ulicina* (Hook. & Arn.) Hook. & Arn.	Asteraceae	Shrub	Endemic	Achene	Wind
*Cistanthe longiscapa* (Barnéoud) Carolin ex Hershk.	Montiaceae	Annual herb	Endemic	Dehiscent capsule	Gravity
*Echinopsis deserticola* (Werderm.) Friedrich & G.D. Rowley	Cactaceae	Succulent shrub	Endemic	Berry	Birds, mammals
*Encelia canescens* Lam.	Asteraceae	Shrub or subshrub	Native	Achene	Wind
*Euphorbia thinophila* Phil.	Euphorbiaceae	Perennial herb	Endemic	Dehiscent capsule	Gravity
*Fagonia chilensis* Hook. & Arn.	Zygophyllaceae	Perennial herb	Native	Capsule	Wind
*Frankenia chilensis* K. Presl	Frankeniaceae	Shrub	Native	Capsule	Wind
*Helenium atacamense* Cabrera	Asteraceae	Annual or biennial herb	Endemic	Achene	Wind
*Heliotropium floridum* (A. DC.) Clos	Heliotropiaceae	Shrub	Endemic	Schizocarps	Water
*Homalocarpus dichotomus* (Poepp. ex DC.) Mathias & Constance	Apiaceae	Annual herb	Endemic	Achene	Wind
*Leucocoryne* sp.	Amaryllidaceae	Perennial herb	Endemic	Dehiscent capsule	Gravity
*Menonvillea linearifolia* (Hook. & Arn.) Al-Shehbaz & Salariato	Brassicaceae	Annual herb	Endemic	Silicle	Wind
*Mesembryanthemum crystallinum* L.	Aizoaceae	Annual herb	Introduced	Capsule	Water
*Nolana divaricata* (Lindl.) I.M. Johnst.	Solanaceae	Subshrub	Endemic	Schizocarps	Wind
*Nolana leptophylla* (Miers) I.M. Johnst.	Solanaceae	Annual herb	Endemic	Schizocarps	Wind
*Nolana parviflora* (Phil.) Phil.	Solanaceae	Annual herb	Endemic	Schizocarps	Wind
*Oenothera coquimbensis* Gay	Onagraceae	Annual herb	Endemic	Dehiscent capsule	Wind
*Plantago litorea* Phil.	Plantaginaceae	Annual herb	Endemic	Capsule	Water
*Quinchamalium chilense* Molina	Schoepfiaceae	Perennial herb	Native	Achene	Gravity
*Solanum trinominum* J.R. Benn.	Solanaceae	Shrub	Endemic	Berry	Birds, mammals
*Tetragonia maritima* Barnéoud	Aizoaceae	Shrub	Endemic	Dry fruit	Water
*Tetragonia ovata* Phil.	Aizoaceae	Annual herb	Endemic	Dry fruit	Water
*Tetragonia pedunculata* Phil.	Aizoaceae	Annual herb	Native	Dry fruit	Water
*Zephyra compacta* C. Ehrh.	Tecophilaeaceae	Perennial herb	Endemic	Capsule	Wind
*Zephyranthes bagnoldii* (Herb.) Nic. García	Amaryllidaceae	Perennial herb	Endemic	Capsule	Wind

**Table 2.** Spatial aggregation indices for plant species recorded along transects. The indices include the variance-to-mean ratio (I), Morisita’s index (Id), Green’s index (G) and the negative binomial parameter (k). The spatial pattern was classified as highly, moderately, weakly aggregated or random/uniform, based on these indices. The asterisk indicates that the interpretation should be taken with caution due to the low number of occurrences of these species in the transects.

Species	No. of occurrences	Mean	Variance	I	Id	G	k	Spatial pattern
*A. werdermannii*	77	3.85	35.08	9.11	3.03	0.0267	0.47	Highly aggregated
*A. hystrix*	63	3.15	4.03	1.28	1.09	0.0014	11.29	Moderately aggregated
*C. glabrata*	4	0.2	0.48	2.42	10	3	0.14	*Highly aggregated
*C. kingii*	2	0.1	0.2	2	20	19	0.1	*Highly aggregated
*C. ulicina*	11	0.55	0.89	1.62	2.18	0.1182	0.88	Moderately aggregated
*C. longiscapa*	290	14.5	42.47	2.93	1.13	0.0004	7.52	Weakly aggregated
*E. deserticola*	6	0.3	0.85	2.84	8	1.4	0.16	Highly aggregated
*E. canescens*	77	3.85	9.4	2.44	1.36	0.0047	2.67	Weakly aggregated
*E. thinophila*	1	0.05	0.05	1	1	0		*Weakly aggregated
*Fagonia chilensis*	1	0.05	0.05	1	1	0		*Weakly aggregated
*Frankenia chilensis*	9	0.45	0.47	1.05	1.11	0.0139	9.62	Moderately aggregated
*H. atacamense*	2	0.1	0.1	0.95	0	-1		*Random/uniform
*H. floridum*	39	1.95	7.84	4.02	2.51	0.0397	0.65	Highly aggregated
*H. dichotomus*	11	0.55	0.79	1.43	1.82	0.0818	1.28	Moderately aggregated
*Leucocoryne* sp.	1	0.05	0.05	1	1	0		*Weakly aggregated
*M. linearifolia*	9	0.45	1	2.22	3.89	0.3611	0.37	Highly aggregated
*M. crystallinum*	5	0.25	0.41	1.63	4	0.75	0.4	*Highly aggregated
*N. divaricata*	29	1.45	2.05	1.41	1.28	0.01	3.5	Moderately aggregated
*N. leptophylla*	4	0.2	0.48	2.42	10	3	0.14	*Highly aggregated
*N. parviflora*	49	2.45	7.94	3.24	1.89	0.0185	1.09	Weakly aggregated
*O. coquimbensis*	38	1.9	2.94	1.55	1.28	0.0076	3.48	Moderately aggregated
*P. litorea*	1	0.05	0.05	1	1	0		*Weakly aggregated
*Q. chilense*	3	0.15	0.24	1.6	1.6	6.67	0.25	*Highly aggregated
*S. trinominum*	1	0.05	0.05	1	1	0		*Weakly aggregated
*T. maritima*	3	0.15	0.24	1.6	1.6	2.8333	2.83	*Highly aggregated
*T. ovata*	6	0.3	0.33	1.09	1.33	0.0667	3.42	Moderately aggregated
*T. pedunculata*	4	0.2	0.27	1.37	1.33	3.42	0.54	*Highly aggregated
*Z. compacta*	67	3.35	4.66	1.39	1.11	0.0017	8.56	Moderately aggregated
*Z. bagnoldii*	6	0.3	0.64	2.14	5.33	0.8667	0.26	Moderately aggregated
