# Supplementary material for: Community composition and spatial aggregation patterns of the endangered endemic plant Menonvillea linearifolia (Brassicaceae) in the Atacama Desert
Source: Biodivers Data J. 2026 Mar 26;14:e185999. doi: 10.3897/BDJ.14.e185999 (PMC13047373; doi:10.3897/BDJ.14.e185999)
Supplement: Supplementary material 1 — Community Composition and Spatial Aggregation Patterns of the Endangered Endemic Plant Menonvillea linearifolia (Brassicaceae) in the Atacama Desert [file bdj-14-e185999-s001.pdf]

Workflow for species accumulation curves

```
# ==========================================================
# Species accumulation / rarefaction from your Excel matrix
# File: "Matriz presencia ausencia.xlsx"  (sheet: "Hoja1")
# Data format in Excel: rows = species, columns = transects (0/1)
# Output: accumulation curve values + figure
# ==========================================================

# Packages
# install.packages(c("readxl", "vegan", "dplyr", "ggplot2"))
library(readxl)
library(vegan)
library(dplyr)
library(ggplot2)

# ------------------------
# 1) Read Excel
# ------------------------
df <- read_excel("Matriz presencia ausencia.xlsx", sheet = "Hoja1")

# Expect first column = species names, remaining columns = Transecto 1..20
species <- df[[1]]
mat_sp_tr <- as.data.frame(df[,-1])

# Ensure numeric 0/1 and replace NA with 0 (if any)
mat_sp_tr[] <- lapply(mat_sp_tr, function(x) as.numeric(as.character(x)))
mat_sp_tr[is.na(mat_sp_tr)] <- 0

# Set rownames as species
rownames(mat_sp_tr) <- species

# ------------------------
# 2) Transpose to transects x species (required by vegan)
# ------------------------
mat_tr_sp <- t(as.matrix(mat_sp_tr))

# Remove empty transects if any (all zeros)
mat_tr_sp <- mat_tr_sp[rowSums(mat_tr_sp) > 0, , drop = FALSE]

# ------------------------
# 3) Species accumulation curve (random resampling of transects)
# ------------------------
set.seed(123)  # reproducibility
```

```r
acc <- specaccum(mat_tr_sp, method = "random", permutations = 1000)

acc_df <- data.frame(
  n_transects = acc$sites,
  richness_mean = acc$richness,
  richness_sd = acc$sd
) %>%
  mutate(
    ci_low = richness_mean - 1.96 * richness_sd,
    ci_high = richness_mean + 1.96 * richness_sd
  )

# -------------------------
# 4) Plot + export
# -------------------------
p <- ggplot(acc_df, aes(x = n_transects, y = richness_mean)) +
  geom_ribbon(aes(ymin = ci_low, ymax = ci_high), alpha = 0.2) +
  geom_line(linewidth = 1) +
  labs(
    x = "Number of transects (sampling effort)",
    y = "Expected species richness",
    title = "Species accumulation curve (random transect resampling, 1000 permutations)"
  ) +
  theme_classic()

ggsave("species_accumulation_curve.png", p, width = 7, height = 5, dpi = 300)
write.csv(acc_df, "species_accumulation_curve_values.csv", row.names = FALSE)

# Optional: last-step increment (asymptote tendency)
delta_last <- tail(acc_df$richness_mean, 1) - tail(acc_df$richness_mean, 2)[1]
delta_last
```

Matrix

Species	T 1	T2	T3	T4	T5	T6	T7	T8	T9	T10	T11	T12	T13	T14	T15	T16	T17	T18	T19	T20
Alstroemeria werdermannii	0	0	0	0	0	0	0	0	0	0	1	1	1	1	1	1	1	1	1	1
Atriplex hystrix	1	1	1	1	1	1	0	1	0	1	1	0	1	1	1	1	1	1	0	1
Chaetanthera glabrata	0	0	0	0	0	0	0	0	0	0	0	0	0	0	0	1	1	0	0	0
Chorizanthe kingii	0	0	0	0	0	0	0	0	0	0	0	1	0	0	0	0	0	0	0	0
Chuquiraga ulicina	0	0	0	0	0	0	0	0	0	0	0	1	1	0	1	1	1	0	1	0
Cistanthe longiscapa	1	1	1	1	1	1	1	1	1	1	1	1	1	1	1	1	1	1	1	1
Echinopsis deserticola	0	0	1	0	0	0	0	1	0	0	0	0	0	0	0	0	0	1	0	0
Encelia canescens	1	0	1	0	1	0	1	1	1	1	1	1	1	1	1	1	1	1	1	1
Euphorbia thinophila	0	0	0	0	1	0	0	0	0	0	0	0	0	0	0	0	0	0	0	0
Fagonia chilensis	0	0	0	0	0	1	0	0	0	0	0	0	0	0	0	0	0	0	0	0
Frankenia chilensis	1	0	0	0	1	1	1	0	1	0	0	0	0	1	0	0	0	1	0	0
Helenium atacamense	0	0	1	1	0	0	0	0	0	0	0	0	0	0	0	0	0	0	0	0
Heliotropium floridum	1	1	1	1	1	1	1	1	1	0	0	0	0	0	0	0	0	0	0	0
Homalocarpus dichotomus	1	1	0	1	1	1	1	0	1	0	0	0	0	0	0	0	0	0	0	0
Leucocoryne sp.	0	0	0	0	0	0	0	0	0	0	0	0	0	0	1	0	0	0	0	0
Menonvillea linearifolia	0	0	0	0	0	0	1	0	0	0	0	0	0	0	0	0	1	1	1	1
Mesembryanthemum crystallinum	1	1	0	0	1	0	0	0	0	0	0	0	0	0	0	0	0	0	0	0
Nolana divaricata	1	1	1	1	0	0	1	1	1	0	1	1	1	1	1	1	1	0	1	0
Nolana leptophylla	1	1	0	0	0	0	0	0	0	0	0	0	0	0	0	0	0	0	0	0
Nolana parviflora	1	1	1	1	1	1	1	1	1	1	0	0	0	0	0	0	1	1	0	1
Oenothera coquimbensis	1	1	1	1	0	1	1	0	0	1	1	1	1	0	1	1	1	0	1	1
Plantago litorea	0	0	0	0	0	0	0	0	0	0	0	0	0	0	1	0	0	0	0	0
Quinchamalium chilense	0	0	0	0	0	0	0	0	0	0	0	0	0	0	0	0	1	0	1	0
Solanum trinominum	0	0	0	0	0	0	0	0	0	0	0	0	0	1	0	0	0	0	0	0
Tetragonia maritima	0	0	0	0	0	0	0	0	0	0	0	0	0	0	1	0	1	0	0	0
Tetragonia ovata	0	1	1	0	0	0	0	0	0	0	0	1	0	0	1	1	0	0	0	0
Tetragonia pedunculata	1	0	0	0	0	0	0	1	0	1	0	0	0	0	0	0	0	0	0	0
Zephyra compacta	1	1	1	1	1	0	1	1	1	1	1	1	1	1	1	1	1	1	1	1
Zephyranthes bagnoldii	0	0	0	0	0	0	0	0	0	0	0	1	1	1	0	0	0	0	0	0
