# Supplementary material for: Community composition and spatial aggregation patterns of the endangered endemic plant Menonvillea linearifolia (Brassicaceae) in the Atacama Desert
Source: Biodivers Data J. 2026 Mar 26;14:e185999. doi: 10.3897/BDJ.14.e185999 (PMC13047373; doi:10.3897/BDJ.14.e185999)
Supplement: Supplementary material 2 — Community Composition and Spatial Aggregation Patterns of the Endangered Endemic Plant Menonvillea linearifolia (Brassicaceae) in the Atacama Desert [file bdj-14-e185999-s002.pdf]

B) Appendix S2 — Script/workflow en R (I, Id, G, k + clasification + asterisk)

```
# ================================================================
# Appendix S2. Spatial aggregation indices (I, Id, G, k)
# From transect-level occurrence counts per species
# ================================================================
# Input file: "Matriz presencia ausencia.xlsx"
# Expected format: rows = species, columns = transects, values = 0/1 or counts
# First column = species name
# Output: CSV table with indices + classification + low-occurrence flag
# ================================================================

# Packages
# install.packages(c("readxl", "dplyr", "readr"))
library(readxl)
library(dplyr)
library(readr)

# -------------------------
# 1) Read Excel matrix
# -------------------------
df <- read_excel("Matriz presencia ausencia.xlsx", sheet = "Hoja1")

species <- df[[1]]
X <- as.data.frame(df[,-1])

# Coerce to numeric, replace NA with 0
X[] <- lapply(X, function(z) as.numeric(as.character(z)))
X[is.na(X)] <- 0
rownames(X) <- species

n <- ncol(X)  # number of transects (samples)

# -------------------------
# 2) Index functions (match manuscript formulas)
# -------------------------

# Variance-to-mean ratio I = s^2 / xbar
I_var_mean <- function(x) {
  xbar <- mean(x)
  s2 <- var(x)
  if (is.na(xbar) || xbar == 0) return(NA_real_)
  s2 / xbar
}
```

```r
# Morisita Id = [n Σ xi(xi-1)] / [X(X-1)]
Id_morisita <- function(x) {
  Xtot <- sum(x)
  if (Xtot <= 1) return(NA_real_)
  (n * sum(x * (x - 1))) / (Xtot * (Xtot - 1))
}

# Green G = (I - 1) / (Σx - 1)
G_green <- function(I, Xtot) {
  if (is.na(I) || is.na(Xtot) || Xtot <= 1) return(NA_real_)
  (I - 1) / (Xtot - 1)
}

# NegBin k = xbar^2 / (s^2 - xbar)
k_negbin <- function(x) {
  xbar <- mean(x)
  s2 <- var(x)
  if (is.na(xbar) || xbar == 0) return(NA_real_)
  denom <- (s2 - xbar)
  if (denom <= 0) return(Inf)  # ~random/uniform => very large k
  (xbar^2) / denom
}

# Classification rules exactly as in manuscript
classify_species <- function(Id, G, k) {
  # Random/uniform condition first
  if (is.na(Id) || Id <= 1) return("random/uniform")

  # If Id > 1, then decide among aggregated categories using G and k thresholds
  if (!is.na(G) && !is.na(k)) {
    if (G > 0.3 && k < 1) return("highly aggregated")
    if (G >= 0.1 && G <= 0.3 && k >= 1 && k <= 8) return("moderately aggregated")
    if (G < 0.1 && k > 8) return("weakly aggregated")
  }

  # If some indices are NA/Inf, fall back to a conservative label
  return("aggregated (unclassified)")
}

# ------------------------
# 3) Compute indices for each species
# ------------------------
out <- lapply(rownames(X), function(sp) {
```

```
  x <- as.numeric(X[sp, ])

  Xtot <- sum(x)
  xbar <- mean(x)
  s2 <- var(x)

  I <- I_var_mean(x)
  Id <- Id_morisita(x)
  G <- G_green(I, Xtot)
  k <- k_negbin(x)

  category <- classify_species(Id, G, k)

  # Low occurrence flag: total occurrences <= 5
  low_occ_flag <- ifelse(Xtot <= 5, "*", "")

  data.frame(
    species = sp,
    n_transects = n,
    total_occurrences = Xtot,
    mean_occ = xbar,
    variance_occ = s2,
    I = I,
    Id = Id,
    G = G,
    k = k,
    category = category,
    low_occ_flag = low_occ_flag
  )
})

out_df <- bind_rows(out)

# -------------------------
# 4) Export
# -------------------------
write_csv(out_df, "spatial_aggregation_indices_and_categories.csv")
```
